# Phenotypic and Genotypic Diversity Among Symbiotic and Non-symbiotic Bacteria Present in Chickpea Nodules in Morocco

**DOI:** 10.3389/fmicb.2019.01885

**Published:** 2019-09-18

**Authors:** Imane Benjelloun, Imane Thami Alami, Allal Douira, Sripada M. Udupa

**Affiliations:** ^1^Department of Microbiology, National Institute of Agronomical Research, Rabat, Morocco; ^2^Department of Biology, Faculty of Sciences, Ibn Tofail University, Kenitra, Morocco; ^3^ICARDA-INRA Cooperative Research Project, International Center for Agricultural Research in the Dry Areas, Rabat, Morocco

**Keywords:** rhizobia, *Cicer arietinum*, endophytic bacteria, MLSA, gamma proteobacteria, beta proteobacteria

## Abstract

Environmental pollution problems and increased demand for green technologies in production are forcing farmers to introduce agricultural practices with a lower impact on the environment. Chickpea (*Cicer arietinum*) in arid and semi-arid environments is frequently affected by harsh environmental stresses such as heat, drought and salinity, which limit its growth and productivity and affect biological nitrogen fixation ability of rhizobia. Climate change had further aggravated these stresses. Inoculation with appropriate stress tolerant rhizobia is necessary for an environmentally friendly and sustainable agricultural production. In this study, endophytic bacteria isolated from chickpea nodules from different soil types and regions in Morocco, were evaluated for their phenotypic and genotypic diversity in order to select the most tolerant ones for further inoculation of this crop. Phenotypic characterization of 135 endophytic bacteria from chickpea nodules showed a wide variability for tolerance to heavy metals and antibiotics, variable response to extreme temperatures, salinity, pH and water stress. 56% of isolates were able to nodulate chickpea. Numerical analysis of rep-PCR results showed that nodulating strains fell into 22 genotypes. Sequencing of 16S rRNA gene of endophytic bacteria from chickpea nodules revealed that 55% of isolated bacteria belong to *Mesorhizobium* genus. Based on MLSA of core genes (*recA, atpD, glnII and dnaK*), tasted strains were distributed into six clades and were closely related to *Mesorhizobium ciceri*, *Mesorhizobium opportunistum, Mesorhizobium qingshengii, and Mesorhizobium plurifarium.* Most of nodulating strains were belonging to a group genetically distinct from reference *Mesorhizobium* species. Three isolates belong to genus *Burkholderia* of the class β- proteobacteria, and 55 other strains belong to the class γ- proteobacteria. Some of the stress tolerant isolates have great potential for further inoculation of chickpea in the arid and semiarid environments to enhance biological nitrogen fixation and productivity in the context of climate change adaptation and mitigation.

## Introduction

Chickpea (*Cicer arietinum*) is a socially and economically important food legume in South Asia, West Asia, North Africa and Mediterranean regions. It ranks second in area and third in production after beans and pea (Muehlbauer and Sarker, 2017). Like other legumes, chickpea plays a major role in sustainable and environmental friendly agriculture. It can establish symbiosis relation with rhizobia able to reduce atmospheric nitrogen into ammonia directly assimilated by plants, which enhances this crop’s and subsequent cereal crops productivity, improves soil fertility by fixing nitrogen at rates of up to 140 kg/ha/year (Flowers et al., 2010), reduces significantly the use of chemical fertilizers and consequently diminishes global warming and water contamination (De la Peña and Pueyo, 2012). Some rhizobial strains were reported able to promote plant growth and development by enhancing phytohormones production and mineral uptake (Karthik et al., 2017).

Chickpea has been considered as a restrictive host for nodulation (Rivas et al., 2007; Laranjo et al., 2008; Suneja et al., 2016). *Mesorhizobium ciceri* (Nour et al., 1994) and *Mesorhizobium mediterraneum* (Nour et al., 1995) have been described as the only two species nodulating this particular legume (Laranjo et al., 2004; Rivas et al., 2007). However, many other studies showed that other species and genomic groups could also nodulate chickpea (Laranjo et al., 2014; Shamseldin et al., 2017; Tena et al., 2017) namely, *M*. *tianshanense* (Rivas et al., 2007), *M. huakuii, M. amorphae (Laranjo et al., 2008)*, and *M. loti* (Maatallah et al., 2002; Rai et al., 2012). Phylogenetic analysis of the symbiosis genes revealed that they share common symbiosis genes (*nifH* and *nodC*) and similar to those carried by *M. ciceri* and *M. mediterraneum*, which suggests a lateral transfer of symbiosis genes across different species (Rivas et al., 2007; Laranjo et al., 2008). Other studies reported *M*. *opportunistum* and *M. muleiense* able to nodulate chickpea (Zhang et al., 2012). Zhang et al. (2018) isolated a novel species from chickpea nodules, for which the name *Mesorhizobium wenxiniae* sp. nov. is proposed. *Ensifer meliloti* isolated from Tunisian soils was reported to be able to induce ineffective nodules formation in chickpea (Ben Romdhane et al., 2007). Bacteria from *Ensifer* genus have also been described as being able to nodulate chickpea crop in Morocco (Maatallah et al., 2002). However, detailed studies on species nodulating chickpea in Morocco are lacking.

Many phenotypic and molecular tools were used to characterize the diversity among rhizobia nodulating chickpea including DNA homology, G + C content, RFLP of *16S rDNA* (Nour et al., 1995; Maatallah et al., 2002; Rai et al., 2012), rep-PCR using ERIC primers (Dudeja and Singh, 2008; Nandwani and Dudeja, 2009) and whole genome sequencing (Haskett et al., 2016). Analysis of *16S rDNA* is one of the most important methods in taxonomy (Weisburg et al., 1991), however it is not sufficient to distinguish closely related species or strains of same species because of the high sequence conservancy (Laranjo et al., 2012). Combination of other genes sequence analysis to *16S rDNA* like sequence analysis of housekeeping genes *dnaK* (Stepkowski et al., 2003), *glnII* (Stepkowski et al., 2005), *atpD* and *recA* (Vinuesa et al., 2005) have been used to elucidate the taxonomic and phylogenetic relationship among those rhizobia with more resolution than the 16S *rDNA* gene (Alexandre et al., 2009; Delamuta et al., 2012; Tong et al., 2018).

In addition to rhizobia, some studies report the presence of non-symbiotic bacteria mainly *Pantoea*, *Serratia*, *Pseudomonas*, *Bacillus*, *Enterobacter*, and *Burkholderia* living inside legume nodules (Vandamme et al., 2002; Benhizia et al., 2004; Laranjo et al., 2014; Xu et al., 2014; Zaheer et al., 2016). Endophytic bacteria may assist nodulation or promote plant growth either by synthesizing phytohormones such as indole-3-acetic acid (IAA) or by promoting nutrition processes such as phosphate solubilization (Kuklinsky-Sobral et al., 2004; Li et al., 2008). They were also reported to promote growth indirectly by protecting the plant against some fungal pathogens either by producing siderophores, antimicrobial metabolites, or by competing for nutrients and/or niches (O’Sullivan and O’Gara, 1992). Several studies report the use of endophytic bacteria as inoculants to promote nodulation, plant growth, and yields (Vessey, 2003; Tilak et al., 2006; Elkoca et al., 2007; Hung et al., 2007; Liu et al., 2010; César Vicario et al., 2015). Sharma et al. (2012) confirm the presence of *Pseudomonas* spp. and *Erwinia* spp. in chickpea nodules in India, whereas results of Zaheer et al. (2016) reported the presence of *Serratia* spp. as plant growth promoting rhizobacteria in root nodules of chickpeas grown in Pakistan soils.

In Morocco, chickpea is cultivated in arid and semi-arid areas where mineral nitrogen deficiency is an important limiting factor for plant growth (Zahran, 1999). Such areas are prone to water stress and salinization (Hungria and Vargas, 2000; Considine et al., 2017). Moreover, those stresses are usually associated with high temperature and long periods of drought, which affect negatively the establishment of functional nitrogen fixing symbiosis and thus the crop production (Lauter et al., 1981; Davey and Simpson, 1990). Climate change further aggravates these stresses. To improve the growth and the yield of chickpea in such environments and for adaptation to climate change, it is necessary to combine stress tolerant cultivars and stress tolerant rhizobia. Nodulation, nitrogen fixation and growth can be improved by inoculation with competitive and stress tolerant rhizobia particularly when local rhizobia are inefficient or absent (De la Peña and Pueyo, 2012). Studies on rhizobial biodiversity are an important approach to select stress tolerant native isolates. Usually such rhizobial populations are more competent and better adapted to local soils than non-native inoculant strains (Rai et al., 2012).

In this end, the present study was initiated with the following objectives: (1) Isolation of indigenous rhizobia and endophytic non-nodulating bacteria from root nodules of chickpea plants sampled from different soil types in the growing regions in Morocco; (2) Evaluation of the sampled bacteria for their phenotypic diversity for tolerance to harsh environmental stresses; and (3) Evaluation of the sampled bacteria for their genotypic diversity using rep-PCR, 16S rDNA and the core genes sequencing. Based on our results, we identified effective and competitive strains tolerant to various abiotic stresses for their potential use as biofertilizers.

## Materials and Methods

### Isolate Collection

**FIGURE 1:**
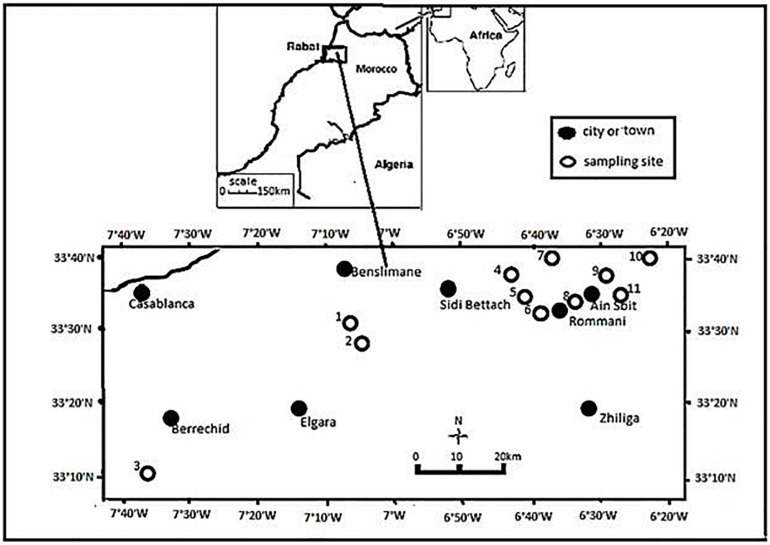
Map showing the sampling sites in Morocco.

In this study, 135 bacterial strains were isolated from chickpea root nodules (Table 1) sampled from fields of chickpea growing areas in Rabat-Sale-Kenitra and Casablanca-Settat regions in Morocco (Figure 1). Sampling fields which have no previous history of inoculation with rhizobia were randomly selected in these two regions and soil samples were collected to characterize pH, electrical conductivity (EC), percentage of organic matter, P_2_O_5_ and K_2_O content.

**TABLE 1:** List of bacterial isolates from root nodules of chickpea sampled in Morocco with soil properties of sampling sites.

		**Mean**	**Mean**										
		**rainfall**	**temperature**										
**Region**	**Site**	**(mm)^a^**	**(°C)^a^**	**Climate**	**Isolates**	**Number of isolates**	**Soil properties**						
							**pH**	**pH**	**P_2_O_5_**	**K_2_O**	**Total**	**Organic**	**EC**
							**(HCl)**	**(H_2_O)**	**(ppm) ^b^**	**(ppm)^b^**	**N (%)**	**matter (%)**	**(ds/m)^c^**
Rabat-Sale-Kenitra	Merchouch 1	449	17.1	Semi-arid	MA- 70, MA-72, MA- 100, MA-101, MA- 121, MA- 122, MA- 124, MA- 125, MA- 127, MA- 140, MA- 146, MA- 147, MA- 148, MA- 151	13	5.7	5.0	15.4	228	0.3	2.2	1.6
Rabat-Sale-Kenitra	Merchouch 2	449	17.1	Semi-arid	MA- 453, MA- 454, MA- 455, MA- 456, MA- 457, MA- 458, MA- 459, MA- 152, MA- 153, MA- 154, MA- 156, MA- 157	12	6.0	5.4	27.8	168	0.3	2.0	1.5
Rabat-Sale-Kenitra	Had Brachoua 1	432	18.0	Semi-arid	MA- 164, MA- 171, MA- 172, MA- 176, MA- 177, MA- 179, MA- 183, MA- 185, MA- 189, MA- 190, MA- 194, MA- 195, MA- 197, MA- 198, MA- 200, MA- 209, MA- 215	17	5.8	4.7	46.7	159	0.3	2.0	1.3
Rabat-Sale-Kenitra	Had Brachoua 2	432	18.0	Semi-arid	MA- 222, MA- 223, MA- 224, MA- 228, MA- 240, MA- 243	6	7.8	7.1	16.5	663	0.3	2.0	2.5
Rabat-Sale-Kenitra	Romani	450	17.4	Semi-arid	MA- 244, MA- 245, MA- 247, MA- 250, MA- 253, MA- 254, MA- 255, MA- 256, MA- 309, MA- 310	10	6.0	5.5	27.8	168	0.4	2.5	1.3
Casablanca-Settat	Benslimane1	401	23.7	Semi-arid	MA- 335, MA- 336, MA- 337, MA- 342, MA- 343, MA- 344, MA- 347, MA- 348, MA- 351, MA- 352	10	8.1	7.1	71.3	714	0.3	1.7	1.6
Rabat-Sale-Kenitra	Ain Sbit	464	17.3	Semi-arid	MA- 355, MA- 356, MA- 358, MA- 361, MA- 362, MA- 364, MA- 365, MA- 367, MA- 369, MA- 371, MA- 373, MA- 374, MA- 375, MA- 381, MA- 382, MA- 383, MA- 384, MA- 387, MA- 388, MA- 389, MA- 390, MA- 391, MA- 392, MA- 394, MA- 396	25	8.0	7.0	10.3	459	0.2	1.5	2.0
Rabat-Sale-Kenitra	Ain Sbit	464	17.3	Semi-arid	MA- 400, MA- 401, MA- 402, MA- 403, MA- 404, MA- 407, MA- 408, MA- 409, MA- 410, MA- 411, MA- 412, MA- 417, MA- 421	13	8.2	7.5	36.5	879	0.3	1.7	1.6
Rabat-Sale-Kenitra	Maaziz	439	17.1	Semi-arid	MA- 422, MA- 423, MA- 424, MA- 425, MA- 426, MA- 427, MA- 428, MA- 429, MA- 430, MA- 431, MA- 432, MA- 433	12	8.3	7.1	4.9	195	0.2	1.5	1.2
Casablanca-Settat	Benslimane2	401	23.7	Semi-arid	MA- 434, MA- 435, MA- 437, MA- 438, MA- 441, MA- 442, MA- 443, MA- 444, MA- 445	9	7.8	6.7	15.4	105	0.3	1.7	4.2
Casablanca-Settat	Sidi El Aidi	361	17.7	Arid	MA- 397, MA- 398, MA- 399, MA- 446, MA- 447, MA- 448, MA- 449, MA- 450	8	8.3	7.1	55.3	960	0.3	1.7	1.3

*^*a*^Average data of 10 years; ^*b*^ppm = mg/kg of soil; ^*c*^Saline soil: EC > 4 ds/m; Normal soil: EC < 4 ds/m.*

Bacterial strains were isolated from surface-sterilized nodules using 70% ethanol and 0.1% HgCl_2_ and washed repeatedly with sterile water. Suspension of crushed sterilized nodules was streaked on yeast extract mannitol (YEM) medium amended with Congo red (Vincent, 1970) using standard procedure (Beck et al., 1993). The purified isolates were conserved in 20% (v/v) glycerol at −80°C.

To make sure that isolated strains were endophytic bacteria and insure good surface sterilization, aliquots of the sterile water used in the final rinse were used to inoculate YEM agar medium. Also, uncrushed surface sterilized nodules were kept in YEM medium plates, inoculated plates were then incubated at 28°C for 7 days and examined for the presence or the absence of microorganisms’ growth.

### Isolates Phenotyping

Except for water stress tolerance, physiological tests were performed on YEM agar plates divided into 16 squares, which were inoculated with 10 μl of bacterial culture (10^8^ cfu/ml grown in YEM broth for 48 h). All treatments were applied with three replications and incubated at 28°C for 7 days.

For temperature tolerance, inoculated plates were incubated at 4, 20, 28, 30, 32, 36, 40, and 44°C.

NaCl tolerance was determined at 0–855 mM NaCl after 7 days of incubation at 28°C (Gao et al., 1994).

Water stress was imposed using 128.4; 188.35; 201.4 and 273.3 g.l^–1^ of polyethylene glycol 6000 (PEG 6000) in YEM broth to reach respectively a level of −0.25; −0.5; −0.75 and −1 MPa (Busse and Bottomley, 1989).

For pH tolerance, pH of YEM media was adjusted using HCl or NaOH for acidic and basic pH, respectively.

To evaluate resistance to intrinsic antibiotics and heavy metals, bacterial cultures were used to inoculate plates with YEM solid media containing filter sterilized antibiotics or heavy metals with the following concentrations: Tested antibiotics were chloramphenicol (25 μg/ml), ampicillin (50 μg/ml), streptomycin (10 μg/ml), spectinomycin (50 μg/ml), kanamycin (50 μg/ml) and tetracycline (25 μg/ml) (Sinclair and Eaglesham, 1984). Tested heavy metals were MnCl_2_ (300 μg/ml), HgCl_2_ (20 μg/ml), CdCl_2_ (20 μg/ml) and ZnCl_2_ (200 μg/ml) (Gao et al., 1994).

### Relations Between Bacterial Strains and Soil From Where They Were Collected

Principal component analysis (PCA) was used to examine relationships between pH and salinity of soils, from where chickpea nodules were collected, and phenotypes (resistance to acid and basic pH and NaCl) of isolated strains.

### Symbiotic Characteristics

To determine the infectivity and effectiveness of isolated strains, bacterial cultures (10^8^ cfu/ml) were used to inoculate surface sterilized seeds of chickpea (1 ml/seed) placed in pots containing sterilized and nitrogen free sand. Once a week, pots were aseptically provided with nitrogen-free solution (CaCl_2_, 1 mM; KH_2_PO_4_, 0.5 mM; ferric citrate, 10 μM; MgSO_4_, 0.25 mM; K_2_S0_4_, 0.25 mM; MnSO_4_, 1 μM; H_3_BO_3_, 2 μM; ZnSO_4_, 0.5 μm; CuSO_4_, 0.2 μm; CoSO_4_, 0.1 μm; Na_2_MoO_4_, 0.1 μM) (Broughton and Dilworth, 1971). The experiment was carried out under controlled conditions with a randomized block design with three replications of each treatment. Two treatments were used as control (T_0_: non-inoculated and non-supplemented with nitrogen and T_N__120_ supplemented with 0.5‰ (w/v) KNO_3_). Plants were irrigated with sterile distilled water every 2 days. Plants were harvested 45 days after planting. Dry shoot biomass and nodules number were determined.

### Genotypic Diversity

Bacterial DNA was extracted using the CTAB method of Saghai-Maroof et al. (1984) with some modifications adapted by Udupa et al. (1999). Quality of isolated DNA was estimated using 1% (w/v) agarose gels.

### Rep-PCR

Genomic fingerprints patterns were obtained for nodulating isolates using rep-PCR method. PCR was performed in a total volume of 25 μl. The reaction mixture contained 9.8 μl of sterile distilled water, 5 μl of 5× PCR buffer (Promega), 1.5 μl of MgCl_2_ (25 mM), 2.5 μl of dNTPs (2 mM) and 0.66 μl of each primer (Rep 1 5′ IIIICGICGICATCIGGC 3′ and Rep 2 5′ ICGICTTATCIGGCCTAC 3′; 0.3 μg each), 2.5 μl of DMSO 100% and 0.4 μl of *Taq* polymerase (Promega; 5 U/μl). 2 μl of DNA (50 ng) was added to this mixture (Versalovic et al., 1991). PCR amplification conditions are detailed in Table 2. Amplified DNA was examined by electrophoresis in agarose gel (1.5%) and visualized under UV using ethidium bromide staining.

**TABLE 2:** List of primers and PCR conditions used in this study.

**Target gene**	**Primers**	**Sequence (5′–3′)**	**PCR conditions**	**References**
*nodC*	nodCF nodCI	AYGTHGTYGAYGACGGTTC CGYGACAGCCANTCKCTATTG	5 min 95°C, 30 × (1 min 95°C, 1 min 55°C, 1.5 min 72°C), 10 min 72°C	Laguerre et al., 2001
*nifH*	nifH-1 nifH-2	AAGTGCGTGGAGTCCGGTGG GTTCGGCAAGCATCTGCTCG	5 min 95°C, 30 × (1 min 95°C, 2 min 62°C, 2 min 72°C), 10 min 72°C	Eardly et al., 1992
*rep* sequence	Rep1 Rep2	IIIICGICGICATCIGGC ICGICTTATCIGGCCTAC	6 min 94°C, 35 × (1 min 94°C, 1 min 40°C, 8 min 65°C), 16 min 65°C	Versalovic et al., 1991

### Amplification of *nodC* and *nifH* Genes

*nodC* and *nifH* genes amplification was performed for all isolates with the intention of confirming the presence of these genes as an indication of their symbiotic potential. PCR amplification was performed in 10 μl reaction mixture containing 1 × PCR buffer (Promega), 1.5 mM of MgCl_2_, 200 μM of each dNTP, 10 pmol of each primer and 0.5 U of *Taq* polymerase (Promega) and 50 ng of DNA. Primers and cycling conditions are detailed in Table 2. PCR products were separated in 1.5% (w/v) agarose gels and visualized under UV using ethidium bromide staining.

### Sequencing of *16S rRNA, recA, atpD, glnII, dnaK*, and *nodC* Genes

Fifty-nine non-nodulating isolates and 46 nodulating isolates belonging to different sites and representative to different rep-PCR and phenotypic clusters were used for *16S rRNA*, *recA, atpD, glnII, dnaK*, and *nodC* gene sequences analysis to identify bacterial isolates from chickpea nodules. Gene sequences of *16S rRNA, atpD, glnII*, and *dnaK* were used from our collaborative study with University of California, Davis, CA, United States on whole genome sequence analysis of the nitrogen-fixing bacterial symbionts of the chickpea (Greenlon et al., 2019). BLAST was used to identify the genes of interest.

### Data Analysis

Comparison of physiological traits was performed as reported by Elboutahiri et al. (2010) on the basis of growth (1) or no growth (0) for each isolate. Comparison of amplified DNA profiles for rep-PCR was performed on the basis of the presence (1) or absence (0) of REP fragments. The binary data was used for estimation of shared allele distance (Jin and Chakraborty, 1994). The shared allele distance was further used for cluster analysis based on UPGMA (unweighted pair group method with arithmetic mean) method using the software program PowerMarker Version 3.25 (Liu and Muse, 2005).

The *16S rDNA, recA, atpD, glnII*, *dnaK*, and *nodC* sequences alignment were performed with MUSCLE^1^. The evolutionary history based on *16S rDNA* and *nodC* genes was inferred by using the Maximum Likelihood method based on the Tamura-Nei model (Tamura and Nei, 1993). The bootstrap consensus tree inferred from 1000 replicates is taken to represent the evolutionary history of the taxa analyzed. The percentage of replicate trees in which the associated taxa clustered together in the bootstrap test (1000 replicates) are shown next to the branches. Evolutionary analyses were conducted in MEGA7 (Kumar et al., 2016).

## Results and Discussion

### Symbiotic Traits

In this study, 135 bacterial isolates were isolated from chickpea nodules and soil properties of sampling sites were analyzed (Table 1). The isolates showed a large diversity in their ability to infect the host and to fix atmospheric nitrogen. Out of 135 isolates, 76 (56%) were able to nodulate chickpea. Number of nodules varied from 3 nodules in the isolate MA-201 to 70 nodules in the isolate MA-72 which was the most infective. Relative effectiveness was estimated by the percentage of each treatment’s shoot dry biomass; MA-100 was the most effective with 86% of the shoot dry weight of the control T_N__120_. The isolates MA-146 and MA-185 were the less effective with 38 and 34% of shoot dry weight respectively of that of control T_N__120_. Shoot dry weight of T_0_ was 28% of the control T_N__120_. Efficient isolates could be further studied under field conditions for chickpea inoculation, particularly those that gave more than 80% of dry shoot biomass as suggested by Maatallah et al. (2002).

### Phenotypic Diversity

**FIGURE 2:**
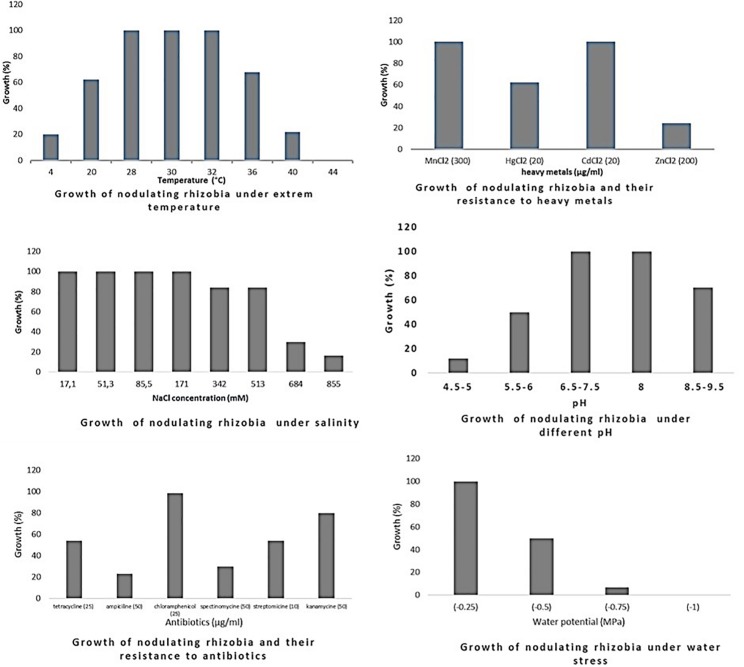
Growth of rhizobia isolated from chickpea nodules under different abiotic stresses. Growth (%) = number of isolates grown (expressed in percentage) at a particular treatment level.

**FIGURE 3:**
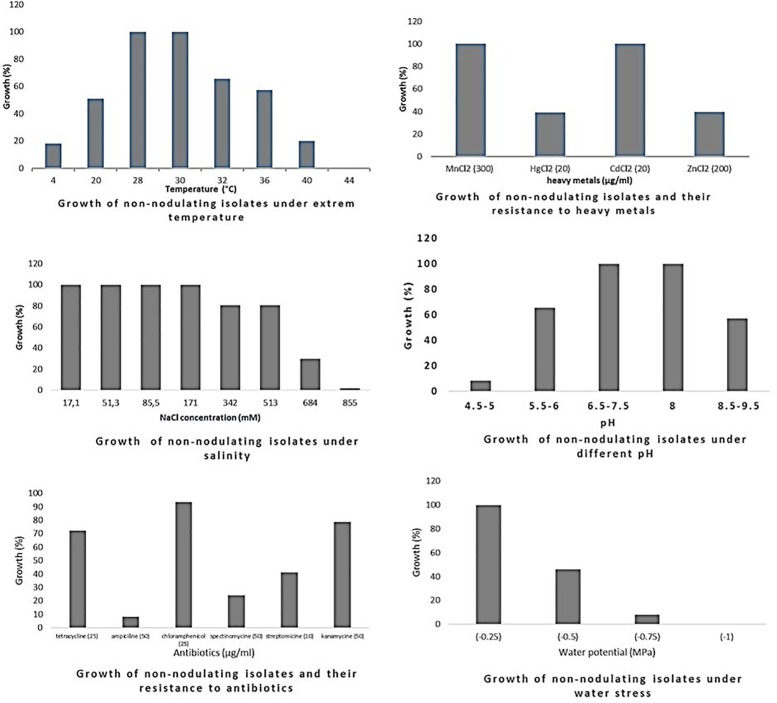
Growth of endophytic non-nodulating bacteria isolated from chickpea nodules under different abiotic stresses. Growth (%) = number of isolates grown (expressed in percentage) at a particular treatment level.

**FIGURE 4:**
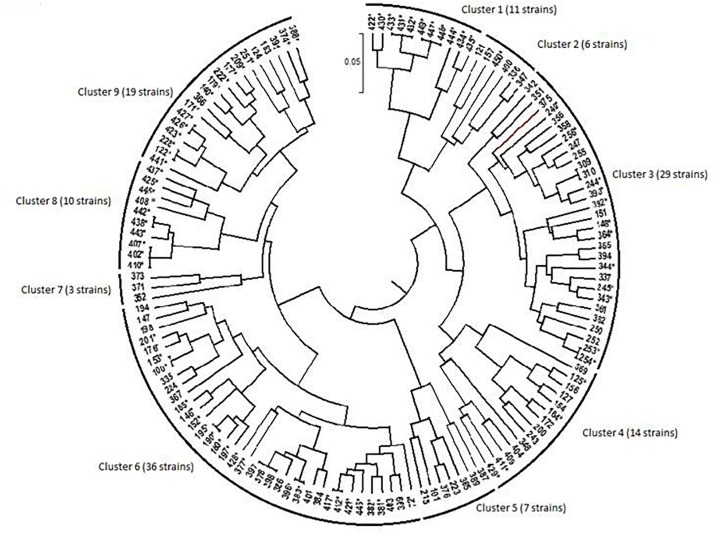
Dendrogram generated by UPGMA clustering method showing relationship among bacterial isolates from chickpea nodules, based on phenotypic variation. The nodulating strains are designed by an asterisk (^∗^). Numbers indicated at the each branch correspond to isolate number that starts with prefix MA-.

Phenotypic diversity among 135 bacterial isolates [76 rhizobia and 59 non-nodulating endophytic bacteria (NNB)] from chickpea nodules was investigated. Phenotypic traits for tolerance to extreme temperatures, pH, salinity and water stress, and resistance to antibiotics and heavy metals showed a large diversity among these isolates. The results are summarized in Table 3, Supplementary Tables S1, S2 and Figures 2–4.

**TABLE 3:** Differentiating phenotypic traits of 135 endophytic bacteria isolated from chickpea nodules.

	**Cluster 1**	**Cluster 2**	**Cluster 3**	**Cluster 4**	**Cluster 5**	**Cluster 6**	**Cluster 7**	**Cluster 8**	**Cluster 9**
**Characteristics**	**(*n* = 11)**	**(*n* = 6)**	**(*n* = 29)**	**(*n* = 14)**	**(*n* = 7)**	**(*n* = 36)**	**(*n* = 3)**	**(*n* = 10)**	**(*n* = 19)**
Infectivity^∗^									
*n* = 3–20	11	1	12	4	0	22	0	10	15
*n* = 21–50	11	1	7	4	0	20	0	7	15
*n* > 50	11	0	0	0	0	17	0	7	10
Relative effectiveness									
34–50%	11	1	12	4	0	22	0	10	15
51–70%	8	0	5	4	0	18	0	5	11
> 70%	4	0	0	0	0	17	0	0	9
Other									
^∗∗^*T*									
*T* = 4°C	0	0	0	5	2	6	1	4	9
*T* = 20°C	0	0	0	7	6	35	2	9	19
*T* = 28°C	11	6	29	14	7	36	3	10	19
*T* = 32°C	11	0	10	14	7	36	3	10	19
*T* = 36°C	0	0	9	7	5	36	0	10	19
*T* = 40°C	0	0	9	0	4	5	0	3	7
*T* = 44°C	0	0	0	0	0	0	0	0	0
NaCl (17.1 mM)	11	6	29	14	7	36	3	10	19
NaCl (51.3 mM)	11	6	29	14	7	36	3	10	19
NaCl (85.5 mM)	11	6	29	14	7	36	3	10	19
NaCl (171 mM)	11	6	29	14	7	36	3	10	19
NaCl (342 mM)	9	0	21	14	0	36	3	10	19
NaCl (513 mM)	7	0	21	14	0	36	3	6	15
NaCl (684 mM)	7	0	8	7	0	1	3	6	8
NaCl (855 mM)	3	0	6	0	0	1	0	3	1
pH 4.5–5	0	0	0	1	0	10	0	0	4
pH 5.5–6	3	6	2	7	0	36	3	2	18
pH 6.5–7.5	11	6	29	14	7	36	3	10	19
pH 8	11	6	29	14	7	36	3	10	19
PH 8.5–9.5	11	3	29	7	1	18	3	10	8
MnCl_2_(300μg/ml)	11	6	29	14	7	36	3	10	19
HgCl_2_ (20 μg/ml)	0	0	0	7	2	31	1	9	19
CdCl_2_ (20 μg/ml)	11	6	29	14	7	36	3	10	19
ZnCl_2_ (200 μg/ml)	0	3	2	14	5	4	1	9	4
^∗∗∗^SH									
SH −0.25 MPa	11	6	29	14	7	36	3	10	19
SH −0.50 MPa	0	0	20	6	0	10	3	9	14
SH −0.75 MPa	0	0	0	0	0	0	0	0	10
SH −1.00 MPa	0	0	0	0	0	0	0	0	0
Tetracycline (25 μg/ml)	7	4	29	4	2	23	0	0	12
Ampicillin (50 μg/ml)	7	0	1	4	0	8	0	0	3
Chloramphenicol (25 μg/ml)	10	4	29	14	6	36	3	10	19
Spectinomycin (50 μg/ml)	4	0	12	4	0	1	2	5	9
Streptomicin (10 μg/ml)	11	0	11	9	0	11	2	5	19
Kanamycin (50 μg/ml)	11	2	20	8	5	36	3	3	19

*^∗^Infectivity: nodules number/plant; ^∗∗^T = Temperature; ^∗∗∗^SH = Water stress.*

**FIGURE 5:**
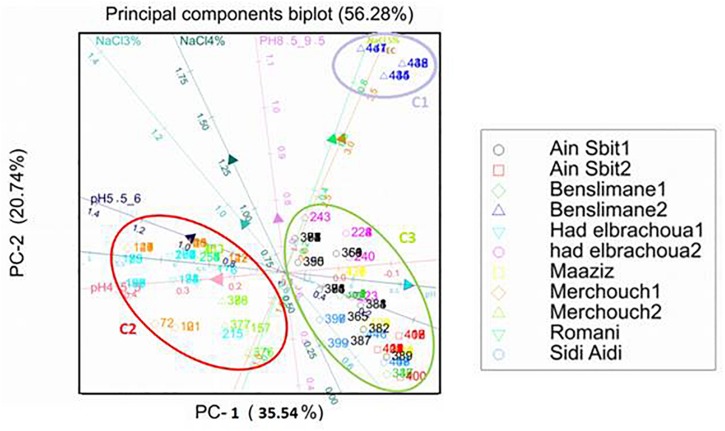
Principal components analysis (PCA): biplot between soil pH and salinity (NaCl concentration) and resistance of bacterial isolates from chickpea nodules to NaCl and to acid and basic pH. Numbers indicated into clusters C1, C2, and C3 correspond to isolate number that starts with prefix MA-.

Optimum growth of all isolates (nodulating and non-nodulating) was observed at pH range of 6–8. 71% of rhizobia and 57% of NNB grew at pH 8.5–9.5. 65% of NNB, and 50% of rhizobia showed good growth at pH 5.5–6 while only 13% of rhizobia and 8% of NNB were able to grow at pH 4.5–5, as they were originated from locations characterized with low pH (4.5–5.5), and none of isolates grew at pH 4. This shows that chickpea endophytic bacteria have a neutral and basic pH tolerance tendency in agreement with other studies on chickpea rhizobia (Nour et al., 1994; Maatallah et al., 2002; Tena et al., 2017). According to Jordan (1982), the pH tolerance range is between 4.5 and 9.5 for *Rhizobiaceae*, however other studies showed that this range may be larger for rhizobia nodulating chickpea, since some isolates grew up to pH 10 (Nour et al., 1994) and pH 4 (Rai et al., 2012). This tolerance to alkaline pH might be related to the basic calcareous nature of soils where chickpea is generally grown in Morocco. This is in contrast to results of Brígido and Oliveira (2013), in which isolates nodulating chickpea showed high sensitivity to pH 9 while 35% of the isolates were acid-sensitive and 45% were highly tolerant to pH 5 or moderately acidophilic. This could be related to the pH of the soil from which chickpea nodules were collected. These results suggest that soil pH contributes to the phenotypic variability that prevails in endophytic bacterial populations in chickpea nodules. The isolates collected from acidic or neutral soils were more resistant to acidic environmental conditions than the ones from alkaline soils (Figure 5), agreeing with several previous reports (Kulkarni and Nautiyal, 1999; Rodrigues et al., 2006; Brígido and Oliveira, 2013). On the other hand, to achieve effective symbiosis the rhizobial symbiont has to deal with the stressful low pH of its leguminous partner that excrete protons and organic acids in the rhizosphere (Marschner et al., 1995). Thus for efficient inoculant with higher symbiotic performance, it was suggested to select strains that tolerate similar conditions of the soil to be cultivated.

The impact of high temperature on microbial metabolism has been reviewed extensively (Frey et al., 2013; Hagerty et al., 2014; Karhu et al., 2014). Climate change such as warming alters microbial soil respiration rates because soil microorganisms and the processes they mediate are temperature sensitive. High soil temperatures affect almost all stages of legume-rhizobia symbiosis (Zahran, 1999; Hungria and Vargas, 2000), and high temperatures influence not only the rhizobial survival in soil, but also the exchange of molecular signals between the two symbiotic partners (Sadowsky, 2005). High temperatures inhibit the adherence of bacteria to root hairs and the formation of root hair and infection thread (Alexandre and Oliveira, 2013). Nodules subjected to salt and drought stress in hot areas revealed a decrease in nitrogen fixation and nitrogenase activity and an increase in fermentative activity (Swaraj and Bishnoi, 1999). In this study, optimum growth of rhizobia nodulating chickpea was observed between 28 and 32°C. 68% grew at 36°C; this percentage decreased to 21% at 40°C and none of the isolates were able to grow at 44°C. Meanwhile, for non-nodulating bacteria (NNB) optimum of growth was between 28 and 30°C and only 65% were able to grow at 32°C and 20% at 40°C. At the lowest temperature of 4°C, 20% of rhizobia and 18% of NNB were able to grow. Most of the isolates identified in this work were thermotolerant as they were isolated from semi-arid regions characterized with high summer temperature, which complements the observation of Eaglesham and Ayanaba (1984) that suggests that sampling from hot areas helps on the selection of high temperature tolerant strains, which is very interesting for developing and applying inoculum for better biological nitrogen fixation. These results were similar to findings of Demissie et al. (2018) on endophytic bacteria present in chickpea nodules in Ethiopia.

Salinity affects rhizobia, endophytic non-nodulating bacteria and host plant inducing ionic stress through the high concentration of ions and osmotic stress due to the change in osmotic concentration around cells causing desiccation and water deficit (De la Peña and Pueyo, 2012). Therefore, selection of salt tolerant isolates seems to be important for chickpea cultivation in salt affected areas. Tolerance of our strains to salinity revealed that there is variable response at a range of 17.1–855 mM of NaCl. All the isolates tolerate until 171 mM of NaCl, 83% of rhizobia and 80% of NNB had good tolerance to 342 mM and to 513 mM NaCl. However, at higher salt concentration the percentage of the tolerant strains decreased to 16% of rhizobia and only 1.6% of NNB grew at 855 mM of NaCl. Tolerant strains were isolated from slightly saline soils, which confirm results obtained by Maatallah et al. (2002) in which 90% of isolates from non-saline soils were inhibited at a level of 342 mM of NaCl and 40% of the isolates from saline soils continued to grow. A similar wide variation in NaCl tolerance by rhizobia nodulating chickpea has been reported by Brígido et al. (2012) and Tena et al. (2017). These results show that a selection pressure for tolerance to salinity is performed by the natural habitat of endophytic bacterial population.

There was a relationship between tolerance to salinity and to water stress, 47% of rhizobia and 46% of NNB grew under water stress of −0.5 MPa. This effect of salt and water stress results in osmotic stress that causes changes in rhizobial morphology (Busse and Bottomley, 1989; Smith and Smith, 1989). The percentage of isolates tolerant to water stress decreases to 7% for rhizobia and 8% for NNB at −0.75 MPa and none grew at −1 MPa. Colonization of plant-beneficial microorganisms is generally decreased by drought, however previous studies indicated that under water stress conditions, inoculation of different chickpea cultivars with rhizobia tolerant to water stress induced an increase in nitrogen fixation and biomass production (Mhadhbi et al., 2008). Evaluation of intrinsic resistance to antibiotics revealed that most of the nodulating and non-nodulating isolates are highly resistant to chloramphenicol and kanamycin, 54% of rhizobia and 69% of NNB grew well with tetracycline. However, only 23% of rhizobia and 8% of NNB exhibited intrinsic resistance to ampicillin. 29% of rhizobia and 25% of NNB were resistant to spectinomycin. The dendrogram in Figure 4 showed that there was no significant relationship concerning antibiotic resistance between isolates clustering and their geographical origin, which is in agreement with findings of Alexandre et al. (2006) while evaluating the natural population of chickpea using antibiotic resistance profiles. Strains with high resistance to different antibiotics could be suggested as potential candidates for chickpea inoculation in areas where antibiotics production by soil microorganisms is frequent. Results from soybean (Anand et al., 2012) showed that inoculation of soybean with an antibiotic and phage-resistant mutant of bradyrhizobia revealed a high ability for nitrogen fixation, thereby increasing soybean production.

The sampled isolates showed good tolerance to heavy metals particularly for cadmium and manganese. 61% of rhizobia and 39% of NNB were able to grow on mercury (HgCl_2_ 20 μg/ml). 25% of rhizobia and 39% of NNB were tolerant to zinc (ZnCl_2_ 200 μg/ml). This is in agreement with results observed by Maatallah et al. (2002) on chickpea rhizobia, which reported that 75% of Moroccan isolates showed good tolerance to mercury and only 20% exhibited a resistance to zinc. That could be related to the nature of the soil from where chickpea nodules were sampled.

The dendrogram obtained from the numerical analysis of nodulating and non-nodulating isolates showed that phenotypic characteristics produced nine clusters at 93% of similarity (Figure 4).

#### Cluster 1

Cluster 1 consisted of 11 nodulating strains from different locations; they were all sensitive to extreme temperatures and acid pH. These strains were tolerant to 342 mM NaCl and to MnCl_2_, CdCl_2_, chloramphenicol, streptomycin and kanamycin_._ Three strains from this cluster were able to grow at 855 mM NaCl.

#### Cluster 2

Cluster 2 consisted of one nodulating strain and five non-nodulating ones from different sites. These isolates were sensitive to extreme temperatures and water stress. Strains of this cluster were tolerant to 171 mM NaCl, MnCl_2_, CdCl_2_.

#### Cluster 3

Cluster 3 consisted of 29 bacterial strains; 12 strains were able to nodulate chickpea. They were all sensitive to low temperatures and acid pH. Most of these strains were tolerant to 513 mM NaCl, MnCl_2_ and CdCl_2_. Nine nodulating strains were able to grow at 40°C and 5 nodulating ones grow at 855 mM NaCl.

#### Cluster 4

Cluster 4 consisted of 14 nodulating and non-nodulating strains from different sites. Most of these strains were able to grow at 513 mM NaCl and at pH range of 6.5–8. These strains were tolerant to chloramphenicol, MnCl_2_, CdCl_2_, and ZnCl_2_. Seven nodulating strains were able to grow at 36°C and 684 mM NaCl.

#### Cluster 5

Cluster 5 consisted of 7 non-nodulating strains able to grow at 171 mM NaCl and pH 8. Strains of this cluster were tolerant to MnCl_2_ and CdCl_2_ and sensitive to ampicillin, spectinomycin and streptomycin.

#### Cluster 6

This is the largest cluster with 36 isolates originating from different sites, only 14 strains were not able to nodulate chickpea. Most of these strains showed good growth at pH range of 5.5–9.5 and in medium supplemented with 171–513 mM NaCl. Bacterial strains of this cluster exhibited resistance to chloramphenicol, kanamycin, MnCl_2_, CdCl_2_, and HgCl_2_ and grew in temperature between 20 and 36°C. Five strains of this cluster were able to grow at 40°C and 6 strains grew at 4°C and pH range of 4.5–5.

#### Cluster 7

Cluster 7 consisted of three non-nodulating strains sensitive to hot temperatures. These isolates grew at pH range of 5.5–9.5. They were resistant to water stress of −0.5 MPa and 684 mM NaCl.

#### Cluster 8

Cluster 8 consisted of 10 nodulating strains tolerant to 342 mM NaCl and pH range of 6.5–9.5. These strains were resistant to chloramphenicol and heavy metals and showed good tolerance to water stress of −0.5 MPa. Three strains of this cluster were tolerant to 855 mM NaCl and 40°C.

#### Cluster 9

Cluster 9 consisted of 19 bacterial strains from different sites, 15 strains from this cluster were able to nodulate chickpea. All the 19 strains were able to grow in temperature between 20 and 36°C, 342 mM NaCl and pH range of 5.5–8. They were all tolerant to streptomycin, kanamycin, chloramphenicol, MnCl_2_, CdCl_2_, and HgCl_2_. 6 nodulating strains from this cluster were able to grow in pH 8.5–9.5 and water stress of −0.75 MPa. One strain could grow at a medium supplemented with 855 mM NaCl.

Phenotypic clusters contained bacterial strains originating from various locations, and strains isolated from the same location belonged to different clusters, which showed the wide phenotypic diversity among these bacterial strains toward multiple environmental stresses. This phenotypic diversity observed in this population could be exploited to select effective and competitive strains tolerant to various abiotic stresses and adapted to marginal edapho-climatic conditions.

Results of principal components analysis between soil pH and salinity and resistance of bacterial isolates to acid and basic pH and NaCl explained 56.28% of the total variance (PC1: 35.55% and PC2: 20.74%) (Figure 5).

According to the length of the arrows and angles among them, we could observe the presence of a soil salinity gradient (EC) which is positively correlated with the soil pH gradient. Those two gradients generated three clusters C1, C2, and C3.

Resistance to 3% NaCl (342 mM) and pH 8.5–9.5 have a slight effect on the distribution of these bacterial strains since the arrows representing them were short.

Resistance to 5% NaCl (855 mM) has a negative correlation with resistance to acid pH, which shows that strains resistant to acid pH were sensitive to high salinity (5% NaCl; cluster C2), while bacterial strains sensitive to acid pH were resistant to high salinity (cluster C1).

Bacterial strains originated from Benslimane 2, characterized with saline soil (EC = 4.2 ds/m), were distributed in cluster C1 which is positively correlated with the positive gradient of soil salinity (EC) and resistance to 5% NaCl.

Bacterial strains originated from Merchouch 1, Merchouch 2, Had Brachoua 1, and Rommani, characterized with acid soil pH and normal soil salinity (EC < 4 ds/m), were distributed in cluster C2 that is positively correlated with resistance to 3% NaCl and pH 4.5–6 and negatively correlated with the positive gradients of soil pH and salinity (EC).

Distribution of isolated bacterial strains in cluster C3 was positively correlated with tolerance to neutral and basic pH and the positive gradient of soil pH, and negatively correlated with the positive gradient of soil salinity (EC) and resistance to 5% NaCl. Bacterial strains of cluster C3 were isolated from Benslimane 1, Had Brachoua 2, Ain Sbit 1, Ain Sbit 2 and Maaziz characterized with neutral to basic soil pH and normal soil salinity (EC < 4 ds/m).

Distribution of isolated strains from chickpea nodules in clusters C1, C2, and C3 showed the positive correlation between strains resistance to pH and salinity (NaCl) and characteristics of soil from where they were isolated. This result shows that a selection pressure for tolerance to acidity and salinity is performed by the natural habitat of isolated bacteria, which suggests that sampling from saline or acid areas helps on the selection of strains tolerant to high salinity or acidity, which is very interesting for developing and applying inoculum for better biological nitrogen fixation.

### Genotyping With Rep-PCR

**FIGURE 6:**
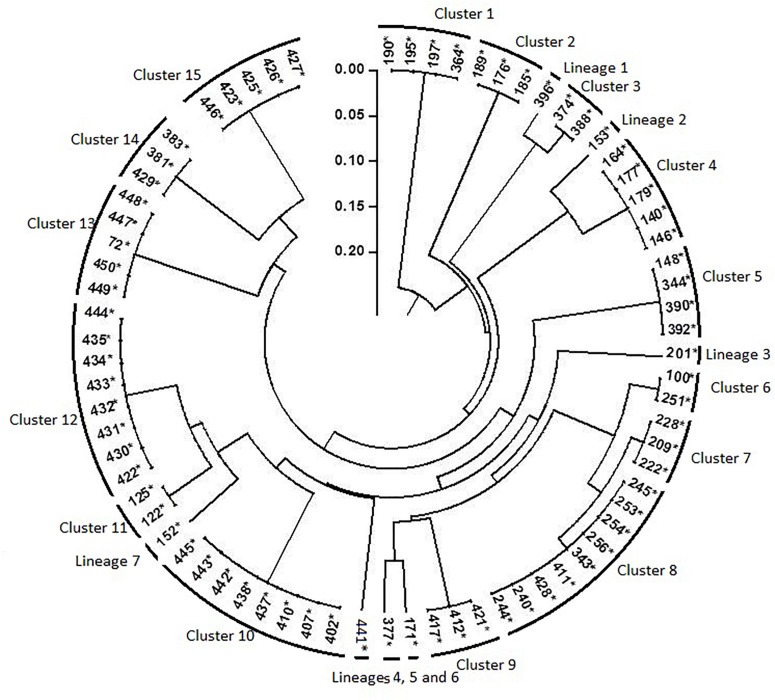
Dendrogram generated by UPGMA clustering method based on results of rep- PCR fingerprinting of 76 rhizobia nodulating chickpea. Numbers indicated at the each branch correspond to isolates number that starts with prefix MA-. The asterisk (^∗^) design nodulating strain.

Results of rep-PCR revealed high diversity among the 76 nodulating strains and classify them into 22 genotypes (15 distinctive clusters and 7 lineages; Figure 6). Each combination of rep-PCR characterizes a unique genotype.

Many rhizobia of a phenotypic cluster were grouped into different clusters of rep-PCR, indicating that isolated rhizobia were highly divergent, even though they belonged to a single phenotypic cluster. There was no relationship between the phenotypic and the genotypic profiles. That could be due to exposure of rhizobia to different soil niches differing in properties as it was reported by Elboutahiri et al. (2010). The evolutionary processes like selection, gene flow/migration, mutation and recombination may have contributed to the evolution of stress tolerant strains, which is observed within the highly divergent isolates. Indeed, recombination or genetic exchange might have played an important role in the generation of a large number of genotypes with similar phenotypes as reported by Provorov and Vorob’ev (2000) and Elboutahiri et al. (2010) in *S. meliloti*. Obtained results are supported by findings of Loureiro et al. (2007) reporting that exposure of soybean rhizobia to stressful tropical environments had increased the number of rep-PCR profiles.

### Analysis of *16S rDNA*, *nodC*, and *Concatenated* Genes *recA, atpD, glnII*, and *dnaK* Sequences

**FIGURE 7:**
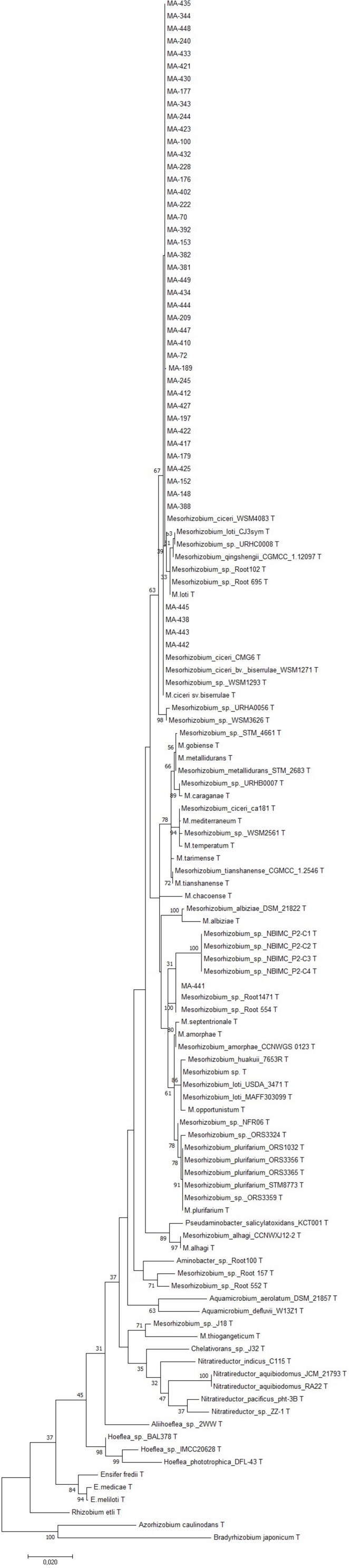
Maximum likelihood phylogenetic tree based on *16S rRNA* gene sequences showing the relationships among chickpea microsymbionts isolates. The bootstrap consensus tree inferred from 1000 replicates to represent the evolutionary history of the taxa analyzed. The percentage of replicate trees in which the associated taxa clustered together in the bootstrap test (1000 replicates) are shown next to the branches.

**FIGURE 8:**
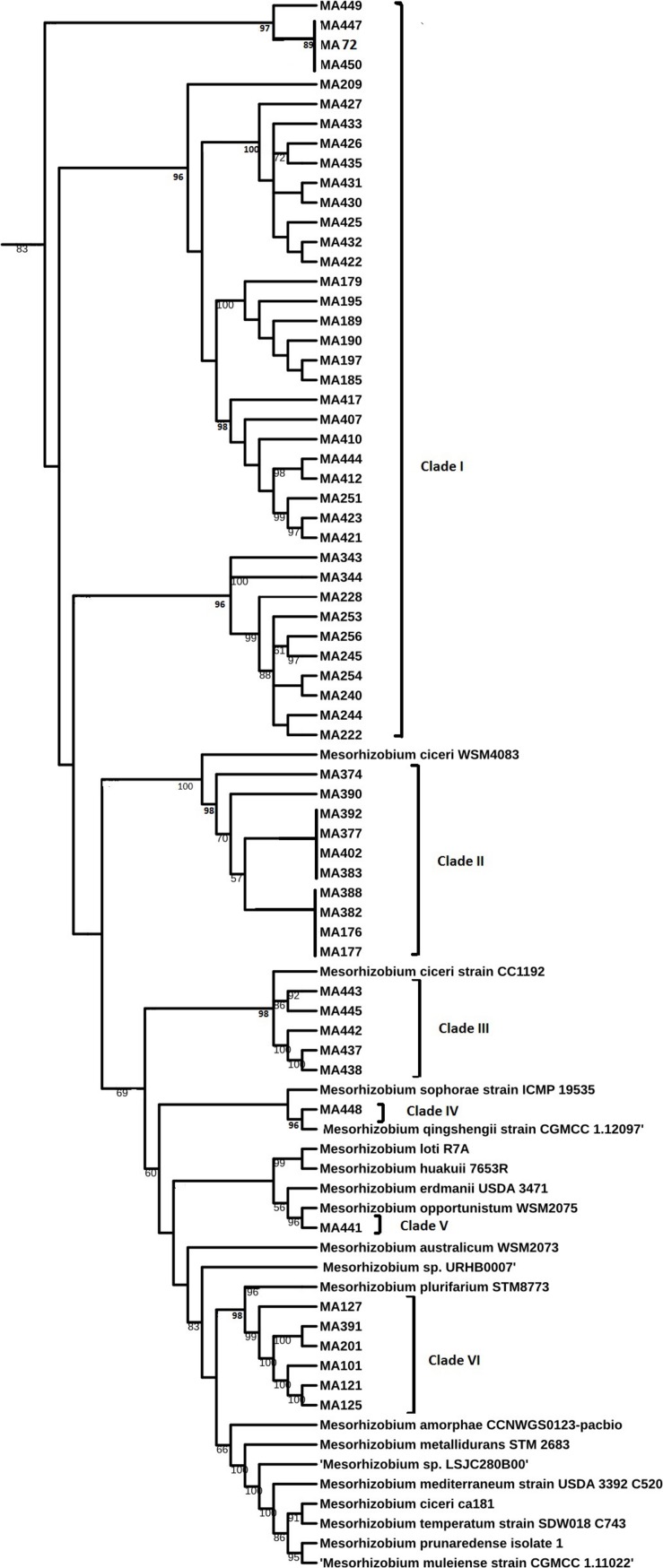
Maximum likelihood phylogenetic tree based on concatenated (*atpD, glnII, recA*, and *dnaK*) gene sequences showing the relationships among chickpea microsymbionts strains, and recognized species of the genus *Mesorhizobium*. The bootstrap consensus tree inferred from 1000 replicates to represent the evolutionary history of the taxa analyzed. The percentage of replicate trees in which the associated taxa clustered together in the bootstrap test (1000 replicates) are shown next to the branches.

**FIGURE 9:**
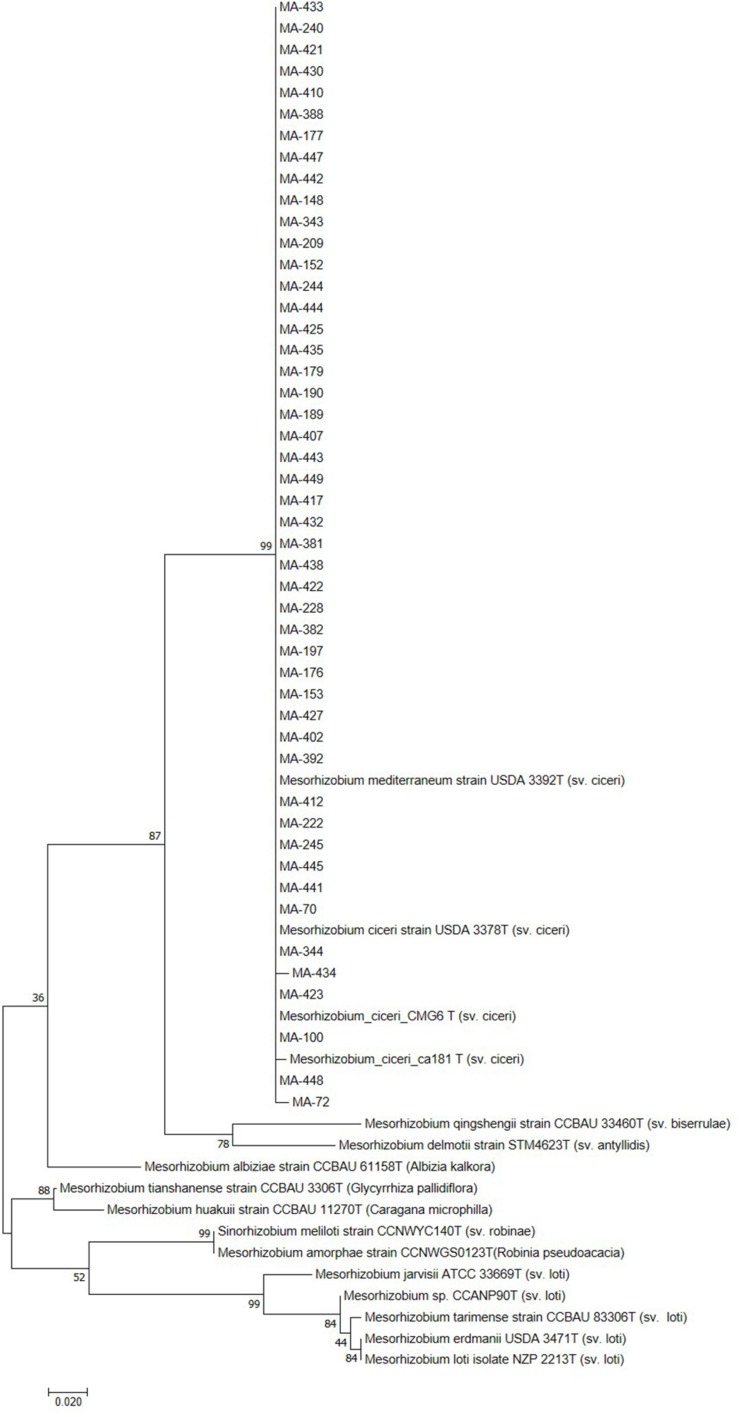
Maximum likelihood phylogenetic tree based on partial sequences of *nodC* gene. The bootstrap consensus tree inferred from 1000 replicates to represent the evolutionary history of the taxa analyzed. The percentage of replicate trees in which the associated taxa clustered together in the bootstrap test (1000 replicates) are shown next to the branches.

Sequencing of *16S rDNA* of 105 retained isolates (46 nodulating strains, belonging to different sites and representative to different rep- and phenotypic clusters and 59 non-nodulating strains) showed that the genus *Mesorhizobium* was the most dominant in all the prospected sites. Obtained sequences were compared with sequences from the gene bank database of different bacterial strains, through the NCBI worldwide website at www.ncbi.nlm.nih.gov/blast. All nodulating strains showed the presence of *nodC* and *nifH* genes. They all belonged to *Mesorhizobium* genus of the family *Rhizobiaceae*. Most of the *Mesorhizobium* isolates showed 99% similarity with *Mesorhizobium cicer*i (Figure 7). Nevertheless, the genus *Mesorhizobium* contains many species with high *16S rRNA* gene sequence identity (Gao et al., 1994; Kwon et al., 2005). Multilocus sequence analysis of core genes found on chromosomes can clarify bacterial relationships better than the analysis of a single locus (Rivas et al., 2009). Phylogenetic tree of concatenated genes (*recA, atpD, glnII*, and *dnaK*) generated from core gene sequence alignments of the test strains and the described reference species (Figure 8), revealed six clusters with high bootstrap support (96 to 100%) at each branch. The different genospecies of different clades belonged to *Mesorhizobium* genus. Genospecies of Clades II and III comprising respectively 10 and 5 strains were found to be closely associated with previously described microsymbiont of chickpea, *M. ciceri* WSM4083 and *M. ciceri* strain CC1192, respectively. Clade IV comprised strain MA-448 that was closely related to the *M. qingshengii* strain CGMCC1.12.97. Clade V contained strain MA-441 that was closely related to type strain *M. opportunistum* WSM2075. The phylogenetic analysis based on concatenated genes identified *M. plurifarium* STM 8773 to be the closest related type strain to the six strains of Clade VI. However, we propose strains of Clade I to be unnamed *Mesorhizobium* genospecies since they formed separate monophyletic groups, distinct from the reference *Mesorhizobium* species. However, sequencing of *nodC* gene of tasted strains revealed high similarity with *nodC* of *M. ciceri* and *M. mediterraneum* (Figure 9) which suggests a lateral transfer of symbiosis genes across different species in agreement with several previous studies (Rivas et al., 2007; Laranjo et al., 2008, 2012).

Previously, *Mesorhizobium ciceri* (Nour et al., 1994) and *Mesorhizobium mediterraneum* (Nour et al., 1995) have been described as the only two species able to establish an effective symbiosis with chickpea (Laranjo et al., 2004; Rivas et al., 2007). However, recent phylogenetic studies on isolates from different geographical locations revealed that other species of *Mesorhizobium* genus may effectively nodulate chickpea (Laranjo et al., 2014; Shamseldin et al., 2017; Tena et al., 2017) including *M*. *tianshanense* (Rivas et al., 2007), *M. huakuii, M. amorphae (Laranjo et al., 2008), M. loti* (Maatallah et al., 2002; Rai et al., 2012), *M*. *opportunistum* and *M. muleiense* (Zhang et al., 2012), and *Mesorhizobium wenxiniae* sp. nov. (Zhang et al., 2018). Findings of Maatallah et al. (2002) while studying diversity of rhizobia nodulating chickpea in Morocco revealed that most rhizobia belonged to the genus *Mesorhizobium*, they were closely related to *M. ciceri, M. loti* and *M. mediterraneum*. In this study, we demonstrated that rhizobia nodulating chickpea in Morocco could be classified with *M. ciceri*, *M. opportunistum*, *M. qingshengii*, *M. plurifarium*, and other unnamed Mesorhizobium genospecies. None of the isolated strains grouped with *M. mediterraneum.*

In this study, three bacteria isolated from inside surface sterilized nodules of chickpea showed 92% similarity with the genus *Burkholderia* of the family *Burkholderiaceae* and class β-proteobacteria. Other bacterial isolates belonged to class γ-proteobacteria. Ten isolates showed 95% similarity with the genus *Pantoea*, 31 isolates showed 97% similarity with the genus *Pseudomonas* and eight isolates showed 97% similarity with the genus *Stenotrophomonas*, five isolates showed 99% similarity with *Serratia* spp. and one isolate 99.8% similarity with *Brevibacterium* sp. One isolate showed 99% similarity with *Bacillus* sp. of the family *Bacillaceae*. This shows that chickpea nodules contain strains other than symbiotic rhizobia, in agreement with finding of Shiraishi et al. (2010) reporting that legumes nodules may contain ‘guest bacteria’ that have no symbiotic ability. These ‘guest bacteria’ may adhere like pathogens or plant growth-promoting bacteria (Egamberdieva et al., 2017). Several recent reviews reported nodulation of other legumes by species of the class gamma and β -proteobacteria (Rivas et al., 2009; Berrada and Fikri Benbrahim, 2014; Shamseldin et al., 2017). Benhizia et al. (2004) presented the first evidence of γ-proteobacteria occupying root nodules of *Leguminosae*, indicating that *16S rDNA* sequence analysis of rhizobia nodulating *Hedysarum carnosum, H. spinosissimum*, and *H. pallidum* were belonging to class γ-proteobacteria and including *Pantoea agglomerans, Enterobacter kobei, Enterobacter cloacae, Leclercia adecarboxylata, Escherichia vulneris*, and *Pseudomonas* sp. Moreover, Shiraishi et al. (2010) reported that *Pseudomonas* sp. of the γ-proteobacteria can nodulate *Robinia pseudoacacia* (black locust). On the other hand 17 species of *Burkholderia* and 2 species of *Ralstonia* belonging to β-proteobacteria were reported to be able to nodulate legumes (Shamseldin et al., 2017). Moulin et al. (2001) reported nodulation of *Macroptilium atropurpureum* by *Burkholderia* species. Other nodulating and nitrogen fixing *Burkholderia* species (*B. caribensis, B. phymatum*, and *B. tuberum*) were isolated from *Mimosa bimucronata* and *Mimosa pigra* (Vandamme et al., 2002; Chen et al., 2003, 2005). Furthermore, *B. phymatum* is highly promiscuous as it can effectively nodulate many important legumes such as *Phaseolus vulgaris* (Gyaneshwar et al., 2011). Studies of Talbi et al. (2010) reported that *Phaseolus vulgaris* was nodulated by *B. phymatum* in Morocco. The presence of *nod* and *nif* genes has been demonstrated in those *Burkholderia* strains by Chen et al. (2005), and their nodulation genes were phylogenetically very related to rhizobia of the class α-proteobacteria which shows a lateral transfer of symbiotic genes in the rhizosphere.

Findings from Sharma et al. (2012) confirm the presence of *Pseudomonas* spp. and *Erwinia* spp. in chickpea nodules in India. The results of Zaheer et al. (2016) reported the presence of *Serratia* spp. as plant growth promoting rhizobacteria in root nodules of chickpeas grown in Pakistan soils.

To our knowledge, this is the first report showing that nodules of *Cicer arietinum* in Morocco contain members of *Pantoea* spp., *Pseudomonas* spp., and *Stenotrophomonas* spp. from the class γ-proteobacteria and *Burkholderia* species from the class β-proteobacteria. Further studies on the ability of these isolates to promote chickpea growth might be of interest.

Nowadays, the need to optimize biological nitrogen fixation is urgent and more important than ever before, given the global concern about the current climate change disturbing environmental properties such temperature, water, air and plant species and causing acidification, salinization and nutrient unbalance in soils, which reduces crop yields and affects profoundly agriculture, soil microbiological properties and interactions between plants and microorganisms (Classen et al., 2015; Considine et al., 2017). All these factors may result in altering the microbial distribution and diversity since soil community members differ in their physiology, temperature sensitivity, and growth rates (Briones et al., 2014; Delgado-Baquerizo et al., 2014; Whitaker et al., 2014). For instance, Plant growth-promoting rhizobacteria (PGPR) regulate ecosystem functions such as nitrogen fixation, nitrification and denitrification. Change in the relative abundance of these microorganisms that regulate specific processes can affect directly regulation of these processes, plant growth and yields.

Adverse climate conditions are inducing adaptation processes in plants and microorganisms, which necessitate selection of adapted plant cultivars. Nevertheless, the use of cultivars not fully adapted to new environmental conditions could be supported by the use of adapted plant growth-promotion, particularly under elevated CO_2_ conditions where nutrients such as nitrogen might be limiting, which require the use of enhanced fertilizer. These bacterial strains respond to warming and other perturbations through resistance, allowed by the microbial trait plasticity, or resilience as they return to their initial composition once the stress had passed (Allison and Martiny, 2008). Mhadhbi et al. (2008) reported that rhizobia have a more important contribution to the variance of symbiotic effectiveness under stress conditions than the used cultivar. Also Compant et al. (2010) reported that some strains of PGPR can grow better at a high temperature than at a low temperature and could be of special interest for application in agriculture exposed to increased temperatures.

Occurrence of these abiotic stresses might increase in the near future as a result of global climate change. To boost crop productivity and alleviate the effects of these stresses, appropriate crop management techniques are imperative such as crop rotation, intercropping and biofertilization. Accordingly and responding to climate fluctuations, development of rhizobia and PGPR collections and selection of stress tolerant bacteria seem to be more important than ever as they can be used as biotechnological tools to support nutrient acquisition, thereby improving yield improvement, adaptation and mitigation to global climate change.

## Conclusion

The present study on endophytic nodulating and non-nodulating bacteria isolated from chickpea nodules in Morocco revealed a phenotypic and genotypic diversity. Rhizobia and non-nodulating bacteria present in chickpea root nodules were almost equal in proportion.

Studied strains revealed variable response to abiotic stresses and showed desirable physiological characteristics such as tolerance to extreme temperatures, pH and salinity and environmental toxicity. These provide a basis for selecting rhizobia or potential plant growth promoting bacteria that can be further used as candidates for formulating appropriate inoculum to improve nitrogen fixation and eventually chickpea yield and soil fertility in stressed areas. Some of isolated strains with high symbiotic effectiveness and efficiency were identified able to grow at a medium supplemented with 684–855 mM NaCl and water stress of −0.5 to −0.75 MPa. These potential salt and drought tolerant strains might be used for exploitation to enhance biological nitrogen fixation and chickpea growth in salt and drought affected areas.

Genotypic characterization showed that all nodulating bacteria belong to the genus *Mesorhizobium*. Multilocus sequence analysis of core genes (*recA, atpD, glnII, and dnaK*) showed that most nodulating strains belonged to an unnamed Mesorhizobium genospecies (clade I). Fifteen strains were related to *M. ciceri* (Clades II and III), six strains were related to *M. plurifarium* (CladeVI) and only one strain was closely related to *M. opportunistum* (Clade V), another strain was closely related to *M. qingshengii* (Clade IV) and none were related to *M. mediterraneum*. Our results indicated that the five genospecies that nodulate chickpea share common symbiosis genes of *M. ciceri* and *M. mediterraneum*. In addition to *Mesorhizobium*, we found non-nodulating species belonging to genus *Burkholderia*, *Pantoea*, *Pseudomonas*, and *Stenotrophomonas* in chickpea nodules. Further studies of the ability of those species to promote chickpea growth might be of interest.

## Author Contributions

IB did the sampling of the isolates, isolated the cultures, performed the phenotyping and genotyping of the isolates, and contributed to the drafting of the manuscript. ITA contributed to the conception and the outline of the study, supervision of the phenotyping, and drafting of the manuscript. AD contributed to the conception of the study and drafting of the manuscript. SU contributed to conception and the outline of the study, supervision of genotyping, data analysis and drafting of the manuscript.

## Conflict of Interest Statement

The authors declare that the research was conducted in the absence of any commercial or financial relationships that could be construed as a potential conflict of interest.

## Footnotes

1 https://www.ebi.ac.uk/Tools/msa/muscle/

## References

[B1] Alexandre A. Brigido C. Laranjo M. Rodrigues S. Oliveira S. (2009). Survey of chickpea rhizobia diversity in Portugal reveals the predominance of species distinct from *Mesorhizobium ciceri* and *Mesorhizobium mediterraneum*. *Microb. Ecol.* 58 930–941. 10.1007/s00248-009-9536-6 19468700

[B2] Alexandre A. Laranjo M. Oliveira S. (2006). Natural populations of chickpea rhizobia evaluated by antibiotic resistance profiles and molecular methods. *Microb. Ecol.* 51 128–136. 10.1007/s00248-005-0085-3 16389465

[B3] Alexandre A. Oliveira S. (2013). Response to temperature stress in rhizobia. *Crit. Rev. Microbiol.* 39 219–228. 10.3109/1040841X.2012.702097 22823534

[B4] Allison S. D. Martiny J. B. (2008). Resistance, resilience, and redundancy in microbial communities. *Proc. Natl. Acad. Sci. U.S.A.* 105 11512–11519. 10.1073/pnas.0801925105 18695234 PMC2556421

[B5] Anand A. Jaiswal S. Dhar B. Vaishampayan A. (2012). Surviving and thriving in terms of symbiotic performance of antibiotic and phage-resistant mutants of *Bradyrhizobium* of soybean [*Glycine max* (L.) merrill]. *Curr. Microbiol.* 65 390–397. 10.1007/s00284-012-0166-8 22735983

[B6] Beck D. P. Materon L. A. Afandi F. (1993). *Practical Rhizobium-Legume Technology Manual.* Aleppo: International Center for Agricultural Research in the Dry Areas (ICARDA).

[B7] Ben Romdhane S. Aouani M. E. Mhamdi R. (2007). Inefficient nodulation of chickpea (*Cicer arietinum L*.) in the arid and Saharan climates in Tunisia by *Sinorhizobium meliloti* biovarmedicaginis. *Ann. Microbiol.* 57 15–19. 10.1007/bf03175044

[B8] Benhizia Y. Benhizia H. Benguedouar A. Muresu R. Giacomini A. Squartini A. (2004). Gamma *proteobacteria* can nodulate legumes of the genus *Hedysarum*. *Syst. Appl. Microbiol.* 27 462–468. 10.1078/0723202041438527 15368852

[B9] Berrada H. Fikri Benbrahim K. (2014). Taxonomy of the rhizobia: current perspectives. *Br. Microbiol. Res. J.* 4 616–639. 10.9734/BMRJ/2014/5635

[B10] Brígido C. Alexandre A. Oliveira S. (2012). Transcriptional analysis of major chaperone genes in salt-tolerant and salt-sensitive *Mesorhizobia*. *Microbiol. Res.* 167 623–629. 10.1016/j.micres.2012.01.006 22364959

[B11] Brígido C. Oliveira S. (2013). Most acid-tolerant chickpea *Mesorhizobia* show induction of major chaperone genes upon acid shock. *Microb. Ecol.* 65 145–153. 10.1007/s00248-012-0098-7 22890730

[B12] Briones M. J. I. McNamara N. P. Poskitt J. Crow S. E. Ostle N. J. (2014). Interactive biotic and abiotic regulators of soil carbon cycling: evidence from controlled climate experiments on peatland and boreal soils. *Glob. Change Biol.* 20 2971–2982. 10.1111/gcb.12585 24687903

[B13] Broughton W. Dilworth M. (1971). Control of leghaemoglobin synthesis in snake beans. *Biochem. J.* 125 1075–1080. 10.1042/bj1251075 5144223 PMC1178271

[B14] Busse M. D. Bottomley P. J. (1989). Growth and nodulation responses of rhizobium *meliloti* to water stress induced by permeating and nonpermeating solutes. *Appl. Environ. Microbiol.* 55 2431–2436. 16348021 10.1128/aem.55.10.2431-2436.1989 PMC203100

[B15] César Vicario J. Gallarato L. Paulucci N. Perrig D. Bueno M. A. Dardanelli M. (2015). “Co-inoculation of legumes with *Azospirillum* and symbiotic rhizobia,” in *Handbook for Azospirillum: Technical Issues and Protocols*, eds Cassán F. Okon Y. Creus C. (Cham: Springer International Publishing), 411–418. 10.1007/978-3-319-06542-7_22

[B16] Chen W.-M. James E. K. Chou J.-H. Sheu S.-Y. Yang S.-Z. Sprent J. I. (2005). β-Rhizobia from *Mimosa pigra*, a newly discovered invasive plant in Taiwan. *New Phytol.* 168 661–675. 10.1111/j.1469-8137.2005.01533.x 16313648

[B17] Chen W. M. Moulin L. Bontemps C. Vandamme P. Béna G. Boivin-Masson C. (2003). Legume symbiotic nitrogen fixation by β-*Proteobacteria* is widespread in nature. *J. Bacteriol.* 185 7266–7272. 10.1128/jb.185.24.7266-7272.2003 14645288 PMC296247

[B18] Classen A. T. Sundqvist M. K. Henning J. A. Newman G. S. Moore J. A. Cregger M. A. (2015). Direct and indirect effects of climate change on soil microbial and soil microbial-plant interactions: what lies ahead? *Ecosphere* 6 1–21.

[B19] Compant S. Sessitsch A. Van Der Heijden M. G. A. (2010). Climate change effects on beneficial plant–microorganism interactions. *FEMS Microbiol. Ecol.* 73 197–214. 10.1111/j.1574-6941.2010.00900.x 20528987

[B20] Considine M. J. Siddique K. H. M. Foyer C. H. (2017). Nature’s pulse power: legumes, food security and climate change. *J. Exp. Bot.* 68 1815–1818. 10.1093/jxb/erx099 28499041 PMC5429020

[B21] Davey A. G. Simpson R. J. (1990). Nitrogen fixation by subterranean clover at varying stages of nodule dehydration: I. carbohydrate status and short-term recovery of nodulated root respiration. *J. Exp. Bot.* 41 1175–1187. 10.1093/jxb/41.9.1175

[B22] De la Peña T. C. Pueyo J. J. (2012). Legumes in the reclamation of marginal soils, from cultivar and inoculant selection to transgenic approaches. *Agron. Sustain. Dev.* 32 65–91. 10.1007/s13593-011-0024-2

[B23] Delamuta J. Ribeiro R. Menna P. Villamil Bangel E. Hungria M. (2012). Multilocus sequence analysis (MLSA) of *Bradyrhizobium* strains: revealing high diversity of tropical diazotrophic symbiotic bacteria. *Braz. J. Microbiol.* 43 698–710. 10.1590/S1517-83822012000200035 24031882 PMC3768805

[B24] Delgado-Baquerizo M. Maestre F. T. Escolar C. Gallardo A. Ochoa V. Gozalo B. (2014). Direct and indirect impacts of climate change on microbial and biocrust communities alter the resistance of the N cycle in a semiarid grassland. *J. Ecol.* 102 1592–1605. 10.1111/1365-2745.12303

[B25] Demissie N. Degefu T. Ergena A. Ojiewo C. (2018). Phenotypic characteristics of rhizobial and non-rhizobial isolates recovered from root nodules of chickpea (*Cicer arietinum L*.) grown in Ethiopia. *Afri. J. Microbiol. Res.* 12 73–85. 10.5897/ajmr2017.8767

[B26] Dudeja S. S. Singh P. (2008). High and low nodulation in relation to molecular diversity of chickpea mesorhizobia in Indian soils. *Arch. Agron. Soil Sci.* 54 109–120. 10.1080/03650340701747005

[B27] Eaglesham A. Ayanaba A. (1984). “Tropical stress ecology of rhizobia, root nodulation and legume fixation,” in *Current Developments in Biological Nitrogen Fixation*, ed. Subba Rao N. S. (London: Edward Arnold Ltd.), 1–35.

[B28] Eardly B. D. Young J. P. Selander R. K. (1992). Phylogenetic position of Rhizobium sp. strain Or 191, a symbiont of both *Medicago sativa* and *Phaseolus vulgaris*, based on partial sequences of the 16S rRNA and nifH genes. *Appl. Environ. Microbiol.* 58 1809–1815. 1377901 10.1128/aem.58.6.1809-1815.1992 PMC195688

[B29] Egamberdieva D. Wirth S. J. Shurigin V. V. Hashem A. Abd Allah E. F. (2017). Endophytic bacteria improve plant growth, symbiotic performance of chickpea (*Cicer arietinum L*.) and induce suppression of root rot caused by *Fusarium solani* under salt stress. *Front. Microbiol.* 8:1887. 10.3389/fmicb.2017.01887 29033922 PMC5625113

[B30] Elboutahiri N. Thami-Alami I. Udupa S. M. (2010). Phenotypic and genetic diversity in *Sinorhizobium meliloti* and *S. medicae* from drought and salt affected regions of Morocco. *BMC Microbiol.* 10:15. 10.1186/1471-2180-10-15 20089174 PMC2823721

[B31] Elkoca E. Kantar F. Sahin F. (2007). Influence of nitrogen fixing and phosphorus solubilizing bacteria on the nodulation, plant growth, and yield of chickpea. *J. Plant Nutr.* 31 157–171. 10.1080/01904160701742097

[B32] Flowers T. J. Gaur P. M. Gowda C. L. Krishnamurthy L. Samineni S. Siddique K. H. (2010). Salt sensitivity in chickpea. *Plant Cell Environ.* 33 490–509. 10.1111/j.1365-3040.2009.02051.x 19843257

[B33] Frey S. D. Lee J. Melillo J. M. Six J. (2013). The temperature response of soil microbial efficiency and its feedback to climate. *Nat. Clim. Change* 3 395–398. 10.1111/gcb.14281 29682861

[B34] Gao J. L. Sun J. G. Li Y. Wang E. T. Chen W. X. (1994). Numerical taxonomy and DNA relatedness of tropical Rhizobia isolated from Hainan Province. *China. Int. J. Syst. Evol. Microbiol.* 44 151–158. 10.1099/00207713-44-1-151

[B35] Greenlon A. Chang P. L. Damtew Z. M. Muleta A. Carrasquilla-Garcia N. Kim D. (2019). Global-level population genomics reveals differential effects of geography and phylogeny on horizontal gene transfer in soil bacteria. *Proc. Natl. Acad. Sci. U.S.A.* 116 15200–15209. 10.1073/pnas.1900056116 31285337 PMC6660780

[B36] Gyaneshwar P. Hirsch A. M. Moulin L. Chen W. M. Elliott G. N. Bontemps C. (2011). Legume-nodulating beta *proteobacteria*: diversity, host range, and future prospects. *Mol. Plant Microbe Interact.* 24 1276–1288. 10.1094/mpmi-06-11-0172 21830951

[B37] Hagerty S. B. Van Groenigen K. J. Allison S. D. Hungate B. A. Schwartz E. Koch G. W. (2014). Accelerated microbial turnover but constant growth efficiency with warming in soil. *Nat. Clim. Change* 4 903–906. 10.1038/nclimate2361

[B38] Haskett T. Wang P. Ramsay J. O’Hara G. Reeve W. Howieson J. (2016). Complete genome sequence of *Mesorhizobium ciceri* Strain CC1192, an efficient nitrogen-fixing microsymbiont of *Cicer arietinum*. *Genome Announc.* 4:e00516-16. 10.1128/genomeA.00516-16 27284135 PMC4901226

[B39] Hung P. Q. Kumar S. M. Govindsamy V. Annapurna K. (2007). Isolation and characterization of endophytic bacteria from wild and cultivated soybean varieties. *Biol. Fert. Soils* 44 155–162. 10.1007/s00374-007-0189-7

[B40] Hungria M. Vargas M. A. T. (2000). Environmental factors affecting N2 fixation in grain legumes in the tropics, with an emphasis on Brazil. *Field Crops Res.* 65 151–164. 10.1016/S0378-4290(99)00084-2

[B41] Jin L. Chakraborty R. (1994). Estimation of genetic distance and coefficient of gene diversity from single-probe multilocus DNA fingerprinting data. *Mol. Biol. Evol.* 11 120–127. 10.1093/oxfordjournals.molbev.a040086 8121280

[B42] Jordan D. C. (1982). NOTES: transfer of Rhizobium *japonicum* Buchanan 1980 to *Bradyrhizobium* gen. nov., a genus of slow-growing, root nodule bacteria from Leguminous plants. *Int. J. Syst. Evol. Microbiol.* 32 136–139. 10.1099/00207713-32-1-136

[B43] Karhu K. Auffret M. D. Dungait J. A. Hopkins D. W. Prosser J. I. Singh B. K. (2014). Temperature sensitivity of soil respiration rates enhanced by microbial community response. *Nature* 513 81–84. 10.1038/nature13604 25186902

[B44] Karthik C. Oves M. Sathya K. Sri Ramkumar V. Arulselvi P. I. (2017). Isolation and characterization of multi-potential Rhizobium strain ND2 and its plant growth-promoting activities under Cr (VI) stress. *Arch. Agron. Soil Sci.* 63 1058–1069. 10.1080/03650340.2016.1261116

[B45] Kuklinsky-Sobral J. Araujo W. L. Mendes R. Geraldi I. O. Pizzirani-Kleiner A. A. Azevedo J. L. (2004). Isolation and characterization of soybean-associated bacteria and their potential for plant growth promotion. *Environ. Microbiol.* 6 1244–1251. 10.1111/j.1462-2920.2004.00658.x 15560822

[B46] Kulkarni S. Nautiyal C. S. (1999). Characterization of high temperature-tolerant rhizobia isolated from *Prosopis juliflora* grown in alkaline soil. *J. Gen. Appl. Microbiol.* 45 213–220. 10.2323/jgam.45.213 12501363

[B47] Kumar S. Stecher G. Tamura K. (2016). MEGA7: molecular evolutionary genetics analysis version 7.0 for Bigger Datasets. *Mol. Biol. Evol.* 33 1870–1874. 10.1093/molbev/msw054 27004904 PMC8210823

[B48] Kwon S.-W. Park J.-Y. Kim J.-S. Kang J.-W. Cho Y.-H. Lim C.-K. (2005). Phylogenetic analysis of the genera *Bradyrhizobium, Mesorhizobium*, Rhizobium and *Sinorhizobium* on the basis of 16S rRNA gene and internally transcribed spacer region sequences. *Int. J. Syst. Evol. Microbiol.* 55 263–270. 10.1099/ijs.0.63097-0 15653885

[B49] Laguerre G. Nour S. M. Macheret V. Sanjuan J. Drouin P. Amarger N. (2001). Classification of rhizobia based on nodC and nifH gene analysis reveals a close phylogenetic relationship among *Phaseolus vulgaris* symbionts. *Microbiology* 147 981–993. 10.1099/00221287-147-4-981 11283294

[B50] Laranjo M. Alexandre A. Oliveira S. (2014). Legume growth-promoting rhizobia: an overview on the *Mesorhizobium* genus. *Microbiol. Res.* 169 2–17. 10.1016/j.micres.2013.09.012 24157054

[B51] Laranjo M. Alexandre A. Rivas R. Velázquez E. Young J. P. W. Oliveira S. (2008). Chickpea rhizobia symbiosis genes are highly conserved across multiple *Mesorhizobium* species. *FEMS Microbiol. Ecol.* 66 391–400. 10.1111/j.1574-6941.2008.00584.x 18795953

[B52] Laranjo M. Machado J. Young J. P. Oliveira S. (2004). High diversity of chickpea *Mesorhizobium* species isolated in a portuguese agricultural region. *FEMS Microbiol. Ecol.* 48 101–107. 10.1016/j.femsec.2003.12.015 19712435

[B53] Laranjo M. Young J. P. W. Oliveira S. (2012). Multilocus sequence analysis reveals multiple symbiovars within *Mesorhizobium* species. *Syst. Appl. Microbiol.* 35 359–367. 10.1016/j.syapm.2012.06.002 22817876

[B54] Lauter D. J. Munns D. N. Clarkin K. L. (1981). Salt response of chickpea as influenced by N supply 1. *Agron. J.* 73 961–966. 10.2134/agronj1981.00021962007300060013x

[B55] Li J. H. Wang E. T. Chen W. F. Chen W. X. (2008). Genetic diversity and potential for promotion of plant growth detected in nodule endophytic bacteria of soybean grown in Heilongjiang province of China. *Soil Biol. Biochem.* 40 238–246. 10.1016/j.soilbio.2007.08.014

[B56] Liu J. Wang E. T. Chen W. X. (2010). Mixture of endophytic *Agrobacterium* and *Sinorhizobium meliloti* strains could induce nonspecific nodulation on some woody legumes. *Arch. Microbiol.* 192 229–234. 10.1007/s00203-010-0543-2 20098981

[B57] Liu K. Muse S. V. (2005). PowerMarker: an integrated analysis environment for genetic marker analysis. *Bioinformatics* 21 2128–2129. 10.1093/bioinformatics/bti282 15705655

[B58] Loureiro M. D. F. Kaschuk G. Alberton O. Hungria M. (2007). Soybean [*Glycine max* (L.) *Merrill*] rhizobial diversity in Brazilian oxisols under various soil, cropping, and inoculation managements. *Biol. Fert. Soils* 43 665–674. 10.1007/s00374-006-0146-x

[B59] Maatallah J. Berraho E. B. Munoz S. Sanjuan J. Lluch C. (2002). Phenotypic and molecular characterization of chickpea rhizobia isolated from different areas of Morocco. *J. App. Microbiol.* 93 531–540. 10.1046/j.1365-2672.2002.01718.x 12234335

[B60] Marschner H. George E. Römheld V. (1995). “Preface to 2nd Edn.” in *Mineral Nutrition of Higher Plants (Second Edition)*, ed. Marschner H. (London: Academic Press).

[B61] Mhadhbi H. Jebara M. Zitoun A. Limam F. Aouani M. E. (2008). Symbiotic effectiveness and response to mannitol-mediated osmotic stress of various chickpea–rhizobia associations. *World J. Microbiol. Biotechnol.* 24 1027–1035. 10.1007/s11274-007-9571-8

[B62] Moulin L. Munive A. Dreyfus B. Boivin-Masson C. (2001). Nodulation of legumes by members of the beta-subclass of *Proteobacteria*. *Nature* 411 948–950. 10.1038/35082070 11418858

[B63] Muehlbauer F. J. Sarker A. (2017). “Economic importance of chickpea: production, value, and world trade,” in *The Chickpea Genome*, eds Varshney R. K. Thudi M. Muehlbauer F. (Cham: Springer International Publishing), 5–12. 10.1007/978-3-319-66117-9_2

[B64] Nandwani R. Dudeja S. S. (2009). Molecular diversity of a native *Mesorhizobial* population of nodulating chickpea (*Cicer arietinum* L.) in Indian soils. *J. Basic Microbiol.* 49 463–470. 10.1002/jobm.200800355 19322836

[B65] Nour S. M. Cleyet-Marel J. C. Beck D. Effosse A. Fernandez M. P. (1994). Genotypic and phenotypic diversity of Rhizobium isolated from chickpea (*Cicer arietinum* L.). *Can. J. Microbiol.* 40 345–354. 10.1139/m94-057 7915190

[B66] Nour S. M. Cleyet-Marel J.-C. Normand P. Fernandez M. P. (1995). Genomic heterogeneity of strains nodulating chickpeas (*Cicer arietinum* L.) and description of Rhizobium mediterraneum sp. nov. *Int. J. Syst. Evol. Microbiol.* 45 640–648. 10.1099/00207713-45-4-640 7547282

[B67] O’Sullivan D. J. O’Gara F. (1992). Traits of fluorescent *Pseudomonas* spp. involved in suppression of plant root pathogens. *Microbiol. Rev.* 56 662–676. 1480114 10.1128/mr.56.4.662-676.1992 PMC372893

[B68] Provorov N. A. Vorob’ev N. I. (2000). Evolutionary genetics of rhizobia: molecular and population aspects. *Genetika* 36 1573–1587. 11190465

[B69] Rai R. Dash P. K. Mohapatra T. Singh A. (2012). Phenotypic and molecular characterization of indigenous rhizobia nodulating chickpea in India. *Indian J. Exp. Biol.* 50 340–350. 22803324

[B70] Rivas R. García-Fraile P. Velázquez E. (2009). Taxonomy of bacteria nodulating legumes. *Microbiol. Insights* 2 51–69. 10.4137/MBI.S3137

[B71] Rivas R. Laranjo M. Mateos P. F. Oliveira S. Martínez-Molina E. Velázquez E. (2007). Strains of *Mesorhizobium amorphae* and *Mesorhizobium tianshanense*, carrying symbiotic genes of common chickpea endosymbiotic species, constitute a novel biovar (ciceri) capable of nodulating *Cicer arietinum*. *Lett. Appl. Microbiol.* 44 412–418. 10.1111/j.1472-765X.2006.02086.x 17397480

[B72] Rodrigues C. S. Laranjo M. Oliveira S. (2006). Effect of heat and pH stress in the growth of chickpea mesorhizobia. *Curr. Microbiol.* 53 1–7. 10.1007/s00284-005-4515-8 16775779

[B73] Sadowsky M. J. (2005). *Soil Stress Factors Influencing Symbiotic Nitrogen Fixation, Nitrogen Fixation in Agriculture, Forestry, Ecology, and the environment. Nitrogen Fixation: Origins, Applications, and Research Progress*, Vol. 4 Dordrecht: Springer.

[B74] Saghai-Maroof M. A. Soliman K. M. Jorgensen R. A. Allard R. W. (1984). Ribosomal DNA spacer-length polymorphisms in barley: mendelian inheritance, chromosomal location, and population dynamics. *Proc. Natl. Acad. Sci. U.S.A.* 81 8014–8018. 10.1073/pnas.81.24.8014 6096873 PMC392284

[B75] Shamseldin A. Abdelkhalek A. Sadowsky M. J. (2017). Recent changes to the classification of symbiotic, nitrogen-fixing, legume-associating bacteria: a review. *Symbiosis* 71 91–109. 10.1007/s13199-016-0462-3 22527400

[B76] Sharma S. Gaur R. K. Choudhary D. K. (2012). Phenetic and functional characterization of endophytic root-nodule bacteria isolated from chickpea (*Cicer arietinum* L.) and mothbean (*Vigna aconitifolia* L.) of arid-and semi-arid regions of Rajasthan. *India. Pak. J. Biol. Sci.* 15 889–894. 10.3923/pjbs.2012.889.894 24205759

[B77] Shiraishi A. Matsushita N. Hougetsu T. (2010). Nodulation in black locust by the Gammaproteobacteria *Pseudomonas* sp. and the Betaproteobacteria *Burkholderia* sp. *Syst. Appl. Microbiol.* 33 269–274. 10.1016/j.syapm.2010.04.005 20542651

[B78] Sinclair M. J. Eaglesham A. R. J. (1984). Intrinsic antibiotic resistance in relation to colony morphology in three populations of West African cowpea rhizobia. *Soil Biol. Biochem.* 16 247–251. 10.1016/0038-0717(84)90009-9

[B79] Smith L. Smith G. (1989). An osmoregulated dipeptide in stressed Rhizobium *meliloti*. *J. Bacteriol.* 171 4714–4717. 10.1128/jb.171.9.4714-4717.1989 2768187 PMC210271

[B80] Stepkowski T. Czaplinska M. Miedzinska K. Moulin L. (2003). The variable part of the dnaK gene as an alternative marker for phylogenetic studies of rhizobia and related alpha *Proteobacteria*. *Syst. Appl. Microbiol.* 26 483–494. 10.1078/072320203770865765 14666974

[B81] Stepkowski T. Moulin L. Krzyzanska A. McInnes A. Law I. J. Howieson J. (2005). European origin of *Bradyrhizobium* populations infecting lupins and serradella in soils of Western Australia and South Africa. *Appl. Environ. Microbiol.* 71 7041–7052. 10.1128/aem.71.11.7041-7052.2005 16269740 PMC1287703

[B82] Suneja P. Dudeja S. S. Dahiya P. (2016). Deciphering the phylogenetic relationships among rhizobia nodulating chickpea: a review. *J. Appl. Biol. Biotechnol.* Vol 4 061–070.

[B83] Swaraj K. Bishnoi N. R. (1999). Effect of salt stress on nodulation and nitrogen fixation in legumes. *Indian J. Exp. Biol.* 37 843–848. 10687277

[B84] Talbi C. Delgado M. J. Girard L. Ramírez-Trujillo A. Caballero-Mellado J. Bedmar E. J. (2010). *Burkholderia phymatum* strains capable of nodulating *Phaseolus vulgaris* are present in Moroccan soils. *Appl. Environ. Microbiol.* 76 4587–4591. 10.1128/aem.02886-09 20472732 PMC2897466

[B85] Tamura K. Nei M. (1993). Estimation of the number of nucleotide substitutions in the control region of mitochondrial DNA in humans and chimpanzees. *Mol. Biol. Evol.* 10 512–526. 10.1093/oxfordjournals.molbev.a040023 8336541

[B86] Tena W. Wolde-Meskel E. Degefu T. Walley F. (2017). Genetic and phenotypic diversity of rhizobia nodulating Chickpea (*Cicer arietinum* L.) in soils from southern and central Ethiopia. *Can. J. Microbiol.* 63 690–707. 10.1139/cjm-2016-0776 28499096

[B87] Tilak K. Ranganayaki N. Manoharachari C. (2006). Synergistic effects of plant-growth promoting rhizobacteria and rhizobium on nodulation and nitrogen fixation by pigeonpea (*Cajanus cajan*). *European J. Soil Sci.* 57 67–71. 10.1111/j.1365-2389.2006.00771.x

[B88] Tong W. Li X. Huo Y. Zhang L. Cao Y. Wang E. (2018). Genomic insight into the taxonomy of Rhizobium genospecies that nodulate *Phaseolus vulgaris*. *Syst. Appl. Microbiol.* 41 300–310. 10.1016/j.syapm.2018.03.001 29576402

[B89] Udupa S. M. Robertson L. D. Weigand F. Baum M. Kahl G. (1999). Allelic variation at (TAA)n microsatellite loci in a world collection of chickpea (*Cicer arietinum* L.) *germplasm*. *Mol. Gen. Genet.* 261 354–363. 10.1007/s004380050976 10102371

[B90] Vandamme P. Goris J. Chen W. M. de Vos P. Willems A. (2002). Burkholderia tuberum sp. nov. and *Burkholderia phymatum* sp. nov., nodulate the roots of tropical legumes. *Syst. Appl. Microbiol.* 25 507–512. 10.1078/07232020260517634 12583710

[B91] Versalovic J. Koeuth T. Lupski J. R. (1991). Distribution of repetitive DNA sequences in eubacteria and application to fingerprinting of bacterial genomes. *Nucleic Acid Res.* 19 6823–6831. 10.1093/nar/19.24.6823 1762913 PMC329316

[B92] Vessey J. K. (2003). Plant growth promoting rhizobacteria as biofertilizers. *Plant Soil* 255 571–586.

[B93] Vincent J. M. (1970). *A Manual for the Practical Study of the Root-Nodule Bacteria. A Manual for the Practical Study of the Root-Nodule Bacteria.* Oxford: Blackwell Scientific Publications.

[B94] Vinuesa P. Leon-Barrios M. Silva C. Willems A. Jarabo-Lorenzo A. Perez-Galdona R. (2005). Bradyrhizobium canariense sp. nov., an acid-tolerant endosymbiont that nodulates endemic genistoid legumes (*Papilionoideae: Genisteae*) from the Canary Islands, along with *Bradyrhizobium japonicum bv*. genistearum, *Bradyrhizobium genospecies* alpha and *Bradyrhizobium genospecies* beta. *Int. J. Syst. Evol. Microbiol.* 55 569–575. 10.1099/ijs.0.63292-0 15774626

[B95] Weisburg W. G. Barns S. M. Pelletier D. A. Lane D. J. (1991). 16S ribosomal DNA amplification for phylogenetic study. *J. Bacteriol.* 173 697–703. 10.1128/jb.173.2.697-703.1991 1987160 PMC207061

[B96] Whitaker J. Ostle N. Nottingham A. T. Ccahuana A. Salinas N. Bardgett R. D. (2014). Microbial community composition explains soil respiration responses to changing carbon inputs along an a ndes-to-a mazon elevation gradient. *J. Ecol.* 102 1058–1071. 10.1111/1365-2745.12247 25520527 PMC4263258

[B97] Xu L. Zhang Y. Wang L. Chen W. Wei G. (2014). Diversity of endophytic bacteria associated with nodules of two indigenous legumes at different altitudes of the Qilian Mountains in China. *Syst. Appl. Microbiol.* 37 457–465. 10.1016/j.syapm.2014.05.009 24985194

[B98] Zaheer A. Mirza B. S. McLean J. E. Yasmin S. Shah T. M. Malik K. A. (2016). Association of plant growth-promoting *Serratia* spp. with the root nodules of chickpea. *Res. Microbiol.* 167 510–520. 10.1016/j.resmic.2016.04.001 27117242

[B99] Zahran H. H. (1999). Rhizobium-legume symbiosis and nitrogen fixation under severe conditions and in an arid climate. *Microbiol. Mol. Biol. Rev.* 63 968–989. 10585971 10.1128/mmbr.63.4.968-989.1999 PMC98982

[B100] Zhang J. Guo C. Chen W. de Lajudie P. Zhang Z. Shang Y. (2018). Mesorhizobium wenxiniae sp. nov., isolated from chickpea (*Cicer arietinum* L.) in China. *Int. J. Syst. Evol. Microbiol.* 68 1930–1936. 10.1099/ijsem.0.002770 29676730

[B101] Zhang J. J. Liu T. Y. Chen W. F. Wang E. T. Sui X. H. Zhang X. X. (2012). Mesorhizobium muleiense sp. nov., nodulating with *Cicer arietinum* L. *Int. J. Syst. Evol. Microbiol.* 62 2737–2742. 10.1099/ijs.0.038265-0 22228663

